# Developing a Gender Framework for Responsive and Adaptive Design in Digital Health (FORWARD) from a review of reviews

**DOI:** 10.1186/s44247-025-00231-y

**Published:** 2025-12-16

**Authors:** Anna Kalbarczyk, Katya Saksena, Michelle Colder Carras, Isis Gomes, Juliet Zon, Xiaohang Zhou, Smisha Agarwal

**Affiliations:** 1 Department of International Health, Johns Hopkins Bloomberg School of Public Health, Baltimore, MD USA; 2 Center for Global Digital Health Innovation, Johns Hopkins Bloomberg School of Public Health, Baltimore, MD USA

**Keywords:** Gender, Equity, Digital health interventions, Implementation, Framework

## Abstract

**Background:**

Despite the increasing use of digital health interventions (DHIs) in low- and middle-income countries (LMICs), gender disparities persist in access, utilization, and outcomes. Barriers to women’s phone ownership and use include affordability, digital literacy, and social norms; these are not only technological, but institutional as reflected in the limited integration of gender equity in national digital health strategies. While the body of evidence on DHI effectiveness is growing how to implement gender equitable digital programs is less clear.

**Methods:**

We conducted an umbrella review of systematic reviews to synthesize gender-related outcomes on DHIs in LMICs. Eligible DHIs included those used by community health workers and client-facing interventions for health communication, self-management, and symptom monitoring. We included only reviews that reported quantitative outcomes with confidence intervals and reported the study design for included studies. We classified interventions along the Gender Equality Continuum. Findings were reported using PRISMA guidelines.

**Results:**

Of the reviews analyzed, only eight (5.5%) explicitly applied a gender lens. Barriers to women’s participation in DHIs included limited phone ownership, financial dependence, and socio-cultural norms restricting access to health information. Gender-responsive interventions that accounted for these challenges were rare. Gender-transformative approaches (which seek to shift harmful gender norms), such as engaging men in health-seeking behaviors, showed promise but remain underutilized and under evaluated. Measurement of gender-related barriers and their influence on outcomes was limited. No studies used validated gender scales.

**Conclusions:**

Based on these findings, we propose the Gender Framework for Responsive and Adaptive Design in Digital Health (FORWARD) to guide implementers in designing DHIs that address gender disparities. Gender FORWARD emphasizes gender-responsive design at individual (i.e. literacy and access), workforce (i.e. usability, interoperability, and supportive supervision), and ecosystem (i.e. capture and use of gender data in decision-making) levels. This framework offers a critical resource for researchers, implementors, and policy makers to improve digital health equity by ensuring interventions are inclusive, accessible, and sustainable across genders.

## Introduction

Globally, mobile phone connectivity has improved significantly, but access still varies considerably by gender. Gender, the socially constructed attributes, expectations, and roles associated with being a woman, man, girl, boy, or gender minority individual, shapes how individuals interact with one another and with society. According to the GSMA Mobile Gender Gap Report, 2023, women in low- and middle-income countries (LMICs) are 7% less likely than men to own a mobile phone, translating to around 84 million women who remain unconnected [1]. Moreover, the gender gap in mobile internet usage is even more pronounced, with women up to 37% less likely to use mobile internet than men [2]. This gap is particularly notable in regions like South Asia and Sub-Saharan Africa, where barriers such as affordability, digital literacy, and social norms limit women’s access to 3G and 4G connectivity [2–4]. Without specific efforts to address these gaps, national digital strategies risk excluding large segments of the population, particularly women.

These disparities are not only technological but institutional, as reflected in the limited integration of gender equity considerations in national digital health strategies. The Global Digital Health Monitor (GDHM) tracks the use of digital technology in health systems globally across 23 indicators in areas such as leadership and governance, strategy and investment, legislation, workforce, interoperability, and infrastructure [5]. GHDM prioritizes gender as an indicator, calling for gender considerations to be accounted for in digital health strategies and digital health governance. Based on the 2023 GDHM report, 48% of the countries do not consider diversity, equity and human rights in their digital health strategy. 57% of countries do not include gender in their strategies or only consider gender on an ad-hoc basis [5].

Inadequate access to mobile devices and connectivity not only hampers the adoption of digital health interventions but also perpetuates existing inequities in health outcomes. The situation is further complicated by a complex interplay of gender-related social and behavioral factors including social norms and expectations around women’s roles and responsibilities. Many women globally lack financial and decision-making autonomy, which can prevent them from seeking or paying for health services, even when such services are accessible digitally [6]. Patriarchal norms around control frequently extend to the ownership and usage of digital devices, limiting women’s access to personal phones and, consequently, to digital health interventions (DHIs) [7, 8]. These challenges are compounded among community health workers, who are often the primary implementers for DHIs at the local level. This workforce is predominantly female and typically has lower levels of literacy and digital literacy than their male counterparts, furthering and exacerbating existing gender inequities within the health system [9]. The design and implementation of DHIs that overlook these gendered dimensions risk reinforcing, rather than bridging, gaps in health equity.

The body of evidence on the effectiveness of DHIs in improving health and equity outcomes is growing across a range of intervention types and geographies [10–12]. However, it remains unclear what works and for whom – that is, how do we implement gender equitable digital programs that consider the unique needs of individuals, particularly those that may have reduced access to these types of technologies? Several digital health frameworks have been developed to guide the design, implementation, and evaluation of DHIs including the WHO Digital Health Strategy Framework, The Design and Evaluation of DHIs (DEDHI) Framework, and Digital Public Health Framework (DigiPHrame) [13, 14]. Such frameworks often overlook gender considerations, potentially leading to missed opportunities to address the unique needs of women, men, girls, and boys and gender minority individuals [15, 16].

Building on the understanding of gender disparities in digital health, it is therefore crucial to explore frameworks that actively address these inequities. Gender transformative approaches offer a powerful lens for reimagining DHIs. These frameworks go beyond merely acknowledging gender differences to actively challenge and transform the root causes of gender inequality. The Gender Integration Continuum, developed by the Interagency Gender Working Group, provides a spectrum from gender-blind to gender-transformative interventions [17]. At the transformative end, programs seek to reshape gender roles and promote more equitable relationships. Similarly, the Gender at Work framework emphasizes the interplay between individual and systemic change, highlighting how interventions can address both visible and hidden aspects of discrimination [18]. Adding an intersectionality lens further helps us recognize multiple, intersecting forms of oppression and marginalization, including gender, race, class, sexuality, and disability [19]. This intersectional lens is particularly relevant in the context of DHIs, where access and engagement may be influenced by a variety of socio-cultural factors.

By incorporating gender transformative principles DHIs have the potential to not only improve health outcomes but also contribute to broader social change. This review aims to synthesize gender-related outcomes from primary studies identified in a broader review of reviews on DHIs in LMICs. We present findings from a focused gender analysis to identify key gaps and opportunities for more equitable digital health programming. This strategy enables a broad synthesis of fragmented literature allowing us to capture diverse gender-related outcomes, illuminate gaps in existing evidence, highlight opportunities for more equitable programming, and address contextual challenges. Based on these insights, we introduce a practical framework to guide the development of DHIs that are both effective and gender-responsive, integrating gender norms, roles, and relations into every stage of design and implementation.

## Methods

### Study design

We conducted an umbrella review of systematic reviews to assess the effects of DHIs used to improve primary healthcare in LMICs. Eligible DHIs included those used by community health workers or other frontline providers in primary care settings, as well as client-facing interventions for health communication, self-management, and symptom monitoring. We reported findings using the Preferred Reporting Items for Systematic Reviews and Meta-analyses (PRISMA) guidelines (see Fig. 2). The review was registered in PROSPERO (CRD42024564774).

This manuscript specifically reports on a gender-focused sub-analysis conducted to identify reviews that applied a gender lens in their reporting, interpretation, and analysis, and to derive a framework to support gender-responsive DHI implementation. A gender lens was defined as any explicit consideration of gender in analysis, interpretation, or reporting of results including the use of sex-disaggregated data in addition to the exploration of gender-based barriers or interventions aimed at addressing inequity. These findings were used to generate a framework designed to guide implementors to consider gender within different DHIs.

### Search strategy

Search strategies were developed and refined with a librarian and calibrated against known relevant reviews (e.g., Cochrane reviews authored by the research team and other experts that meet our inclusion and exclusion criteria). The final search strategy was reviewed with a librarian to ensure comprehensive coverage and sound structure. We searched MEDLINE (through PubMed), Prospero, and the Cochrane Database of Systematic Reviews to identify relevant reviews, primary studies, and protocols, whether published or unpublished. From relevant protocols retrieved from Prospero and PubMed, we searched Google, Google Scholar and PubMed to see if a completed review manuscript was available.

Searches were conducted between May 21, 2024, and September 2024. We included publications in all languages that were published from January 2012 onwards. The 2012 cutoff was determined due to the convergence of several key developments in technology, policy, and global health priorities around that time. This includes the proliferation of smartphones and mobile devices which were becoming more affordable and widely used globally, enabling the piloting of DHIs. The rapid expansion of internet infrastructure made digital tools more viable for health communication, data collection, and service delivery, and the subsequent evaluation of these technologies.

We opened a call for evidence that invited unpublished studies or those currently in the process of publication. This call was shared through social media platforms (LinkedIn) and with the Global Digital Health Network (GDHN). We also invited peer-reviewed publications that had a systematic study design and were published after 2020. We re-conducted the search in PubMed to capture newer evidence that would not have been included in systematic reviews; primary studies published after 2019 and indexed in PubMed were included.

### Inclusion and exclusion criteria

We included high-quality reviews of DHIs in primary healthcare in LMICs in any language published from 2012 until July 2024. We included only reviews that reported quantitative outcomes with confidence intervals and reported the study design for included studies. Studies were excluded if they were published before 2012, focused on interventions other than digital interventions, focused on program areas outside of primary health care, or lacked outcomes relevant to our research question. We excluded reviews focused primary on high-income countries (HICs), unless they were published after 2019 and addressed novel interventions that may not yet have diffused to LMICs. We captured recent innovations that may not yet be included in systematic reviews by supplementing the dataset with primary studies published after 2019.

In this gender analysis, reviews and primary studies were only included if a gender lens was reported in the analysis or interpretation of results.

### Screening and data extraction

To improve the efficiency of the screening process, the search results (for the systematic reviews and primary studies) were uploaded to a web-based tool, PICO Portal (www.picoportal.net). The platform uses machine learning to sort the citations and present those citations most likely to be included in full-text screening first. For the calibration of the tool, the first 10% of the citations were screened by 2 team members and disagreements were resolved through discussion. Thereafter, one screener was involved until the recall of rate of citations reached 95%.

All included articles were reviewed for the incorporation of a gender lens – a gender specialist (author AK) provided guidelines to reviewers. Articles were tagged for gender by reviewers; these tagged articles were then thoroughly reviewed by AK and included in the final gender analysis if they explored gender equity in design and implementation of DHIs, measured a gender variable, or explored an impact on gender.

### Gender analysis approach

**Fig. 1:**
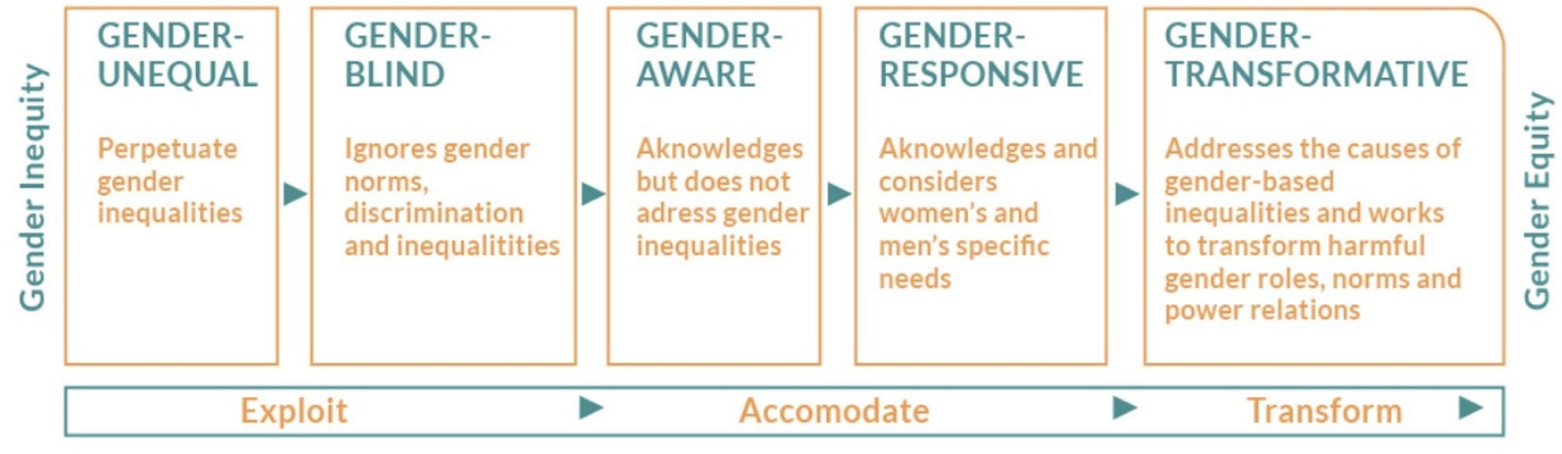
Gender equality continuum. (Adapted from UNFPA, UNICEF and UN Women, 2020)

Gender data was then extracted to identify gender barriers, gender measures, gender outcomes, and descriptions of interventions designed to address gender issues. We used the Gender Equality Continuum adapted from UNFPA, UNICEF, and UN Women (see Fig. 1) to categorize DHI interventions and recommendations.

We classified interventions as gender-blind, gender-sensitive, gender-responsive, or gender-transformative using criteria adapted from the Gender Equality Continuum. For example, interventions that acknowledged gender barriers without addressing them were considered gender-sensitive, while those engaging both men and women to shift norms were classified as gender-transformative.

### Framework development

We used the World Health Organization Classification of digital interventions, services, and applications in health to organize our results in the resulting framework by target - individuals, healthcare workforce, and the data ecosystem [20]. The team then mapped findings from the review to these targets and collaboratively identified linkages between each target area and related findings.

### Quality assessment

We did not assess the methodological quality of the systematic reviews and gender tagging was applied before the study team applied a ‘reliability criterion’ to the reviews. An adapted Newcastle-Ottawa scale was used to determine the quality of studies identified via the primary study search. However, none of these studies were included in the final gender analysis and therefore we do not report on quality assessments in this manuscript.

## Results

### Study selection and characteristics

**Fig. 2:**
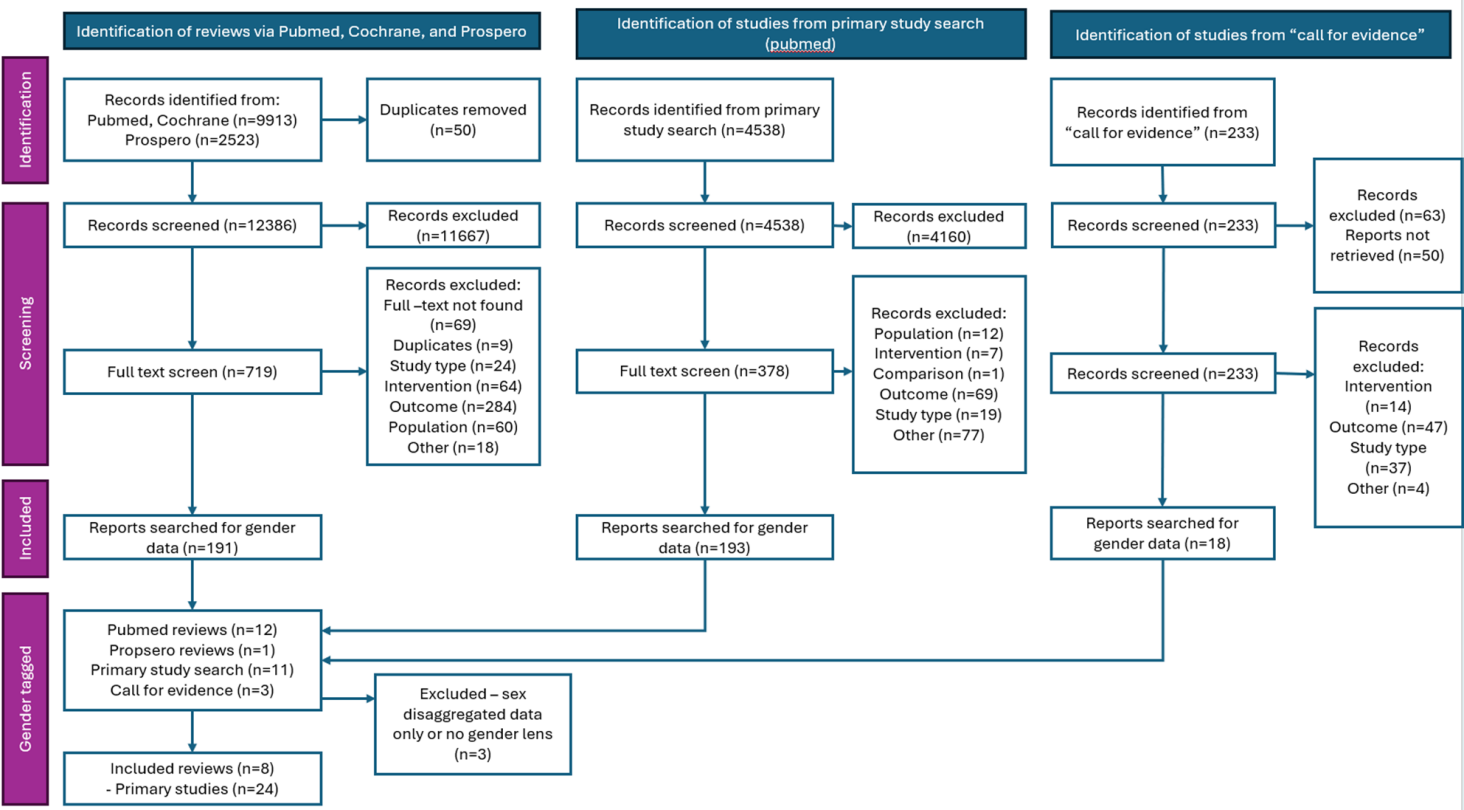
PRISMA diagram

A total of 12,436 systematic reviews, 4538 primary studies and 233 responses in the ‘call for evidence’ were screened for inclusion in the broader review. Of these, 13 systematic reviews, 11 primary studies and 3 studies from the ‘call for evidence’ were tagged for inclusion in the gender analysis. Following review, none of the primary studies were included in the analysis. Eight reviews were included in the gender analysis and twenty-four relevant primary articles were extracted from 6 of those studies. Data are also reported on results and conclusions from 2 reviews which had relevant insights. Figure 2 shows the PRISMA flow diagram of the selection and review process.

### Geographical and topical scope

**Fig. 3:**
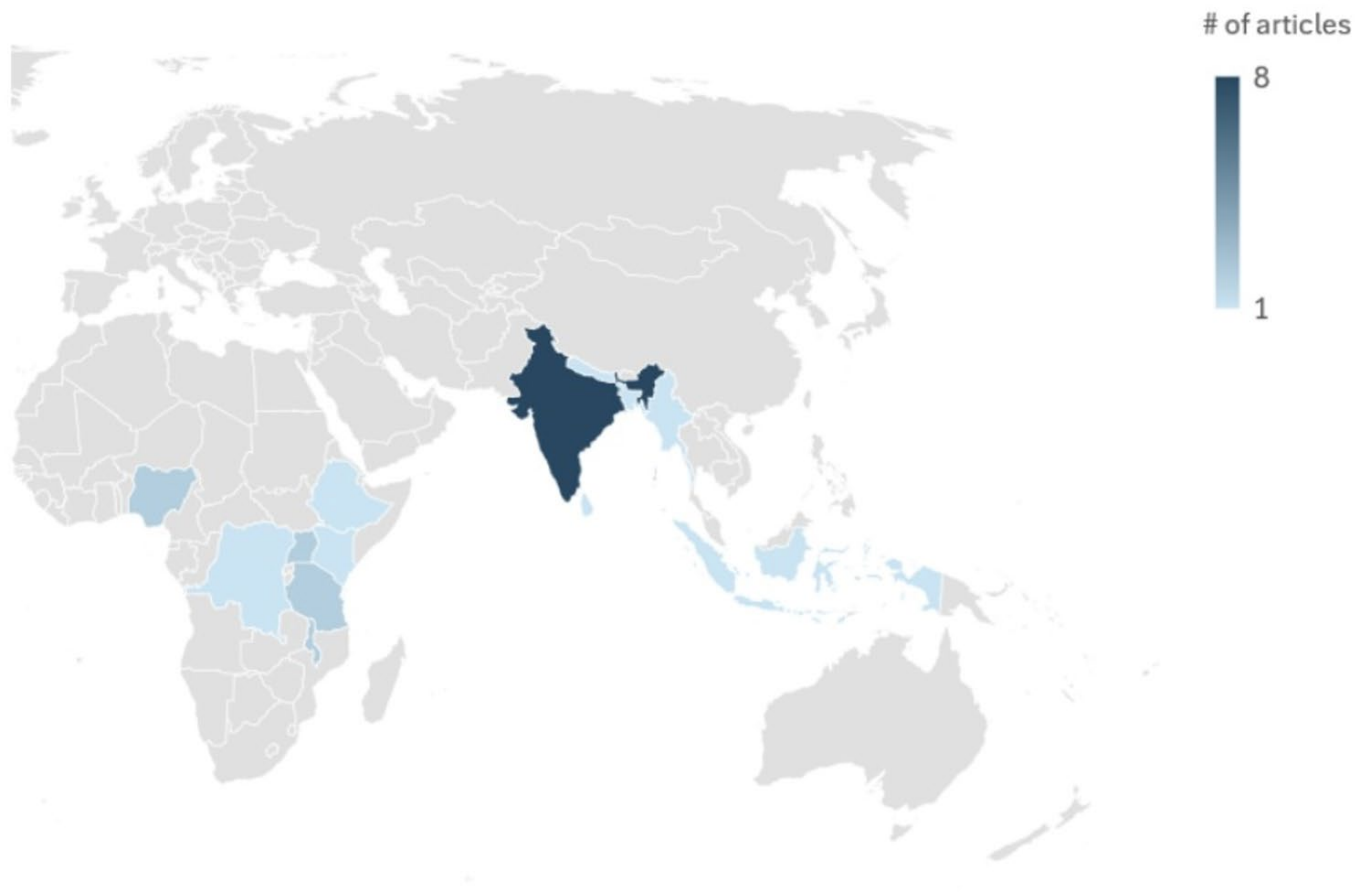
Map of countries included in the study

The 24 primary articles covered a range of topics including maternal and child health (*n* = 6), family planning (*n* = 5), children’s nutrition (*n* = 3), childhood immunization (*n* = 2), adolescent sexual health (*n* = 2), exclusive breastfeeding (*n* = 1), condom use (*n* = 1), HIV/AIDS (*n* = 1), and dengue fever (*n* = 1). Most DHIs were client facing only (*n* = 18), followed by provider facing (*n* = 5); one study explored both client and provider-facing interventions (*n* = 1). Figure 3 displays the countries where studies were conducted. India was most common (*n* = 8), followed by Malawi (*n* = 2), Nigeria (*n* = 2), Tanzania (*n* = 2), and Uganda (*n* = 2). Other studies were conducted in Democratic Republic of Congo (*n* = 1), Bangladesh (*n* = 1), Ethiopia (*n* = 1), Indonesia (*n* = 1), Kenya (*n* = 1), Myanmar (*n* = 1), Nepal (*n* = 1), and Sri Lanka (*n* = 1).

### ^Gender barriers in community and household contexts^

Across articles, the exploration of gender was largely limited to gender-related barriers, which have been presented by individual, household, community, and health system level in Table 1.

**Table 1:** Documented gender barriers

	Gender Barriers
Individual Level	- Cost of transportation services (i.e. when being referred) or engaging with DHIs (i.e. cost of minutes / internet) (*n* = 4). - Women’s low literacy affects text-based DHIs. (*n* = 3) - Preference for women’s vs. men’s voices to deliver sensitive messages. (*n* = 1)
Household Level	- Women’s differential access to phones and phone ownership. (*n* = 9) - Phone sharing with / control by husbands. (*n* = 3) - Husband disapproval of women’s phone use and engagement with DHIs. (*n* = 2) - Women’s agency related to decision-making. (*n* = 2) - Women’s financial dependence on husbands. (*n* = 1)
Community Level	- Socio-cultural beliefs/expectations of young girls, dictating appropriate messaging. (*n* = 1)
Health System and Health Workforce Level	- Gender discrimination in health care services. (*n* = 1) - Increased workload expectations. (*n* = 2) - Challenges adapting to new technology. (*n* = 3)

The most frequently reported barrier was women’s access to phones and phone ownership (*n* = 9) [21–29]. Several studies reported that women had to share phones with their husbands and that husbands controlled women’s phone usage and access to health messages [21, 24, 25]. When husbands control phones, health messages are not always shared or discussed, further limiting women’s access to information [24].

Other barriers included husband disapproval of phone use and engagement with DHIs [23, 30], financial dependence on husbands [25] and restricted use of phones to access sensitive health information (i.e. about sexual health) [21]. In the home, studies identified additional gender barriers related to women’s workload, financial independence, household power dynamics, and transportation / mobility [31]. The cost of transportation and of services themselves were important concerns identified in four articles [21, 27, 31, 32]. Even when DHIs successfully promoted facility-based deliveries, structural barriers like transportation and service costs prevented uptake.

Low literacy among women was a common barrier to effective use of text-based DHIs (*n* = 3) [22, 33, 34], prompting several studies to explore alternatives such as voice messaging [26, 27, 35]. One study using voice-based DHIs explored gendered-preferences for women’s vs. men’s voices to deliver material particularly where literacy was a concern; results indicated preferences for women’s voice narration over men’s narration [35].

Other barriers included women’s agency (related to decision-making, access, etc.) [27, 34], and socio-cultural beliefs and expectations of young girls [21] which dictated what messaging was seen as appropriate.

### Gender barriers in health systems and health workforce

One study identified excessive gender discrimination in health care access by women and stressed the importance of access to non-judgmental and gender-affirming SRH services [36]. The review in which that study was included described how some community members felt health workers were judgmental towards women accessing SRH information [37].

At the health worker level, two studies conducted in India reported that female lady health workers (FLHWs) perceived difficulties adapting to newer technologies and faced increased job responsibilities while having to serve in an already overburdened health system [38, 39]. One study found that uptake of the DHI among staff was less than expected because it was not mainstreamed into the public health system, staff faced numerous technology problems, and staff were concerned that the digitization of incentive calculations might take away flexibility and reduce the total amount of incentive [38].

### Measurement gaps

Measurement of gender-related barriers and their influence on outcomes was limited. None of the studies used validated barrier scales (such as the Digital Intervention Barriers Scale, DIBS-7) or gender indices.

Only one study measured the effects of gender norms on health and behavior outcomes. The researchers assessed the influence of gender norms, exposure to health-related radio programs, interpersonal communication, and social capital on family planning behavior in Uganda [40]. The results of this study’s hierarchical linear modeling showed that all four variables were significant predictors of family planning behavior and that gender norms significantly interacted with individual-level perceived benefits.

One review noted that many studies had limitations in their samples and disaggregation related to gender [36]. Authors indicated that most papers included female participants only and none focused on lesbian, gay, bisexual, transgender, intersex, or queer youth. The authors conclude that “All people who are capable of becoming pregnant…need family planning and abortion care.”

### Gender responsive and transformative programming

One article indicated that women and men and girls and boys use services differently. In Kinshasa, a radio program responded in real-time to anonymous phone call inquiries about sex and related concerns. A survey conducted among 14–24-year-olds who used these calls, found that girls inquired on menstrual cycles calculation, sexual practices, love relationships, and virginity while boys asked questions about masturbation, sexual practices, love relationships, and infections [41].

### Application of the gender equality continuum to DHIs

Using the gender equality continuum (Fig. 1), we categorized DHIs as gender blind, gender responsive, or gender transformative. Most DHIs and related recommendations were *gender blind*, ignoring gender norms, discrimination, and inequalities.

*Gender responsive* interventions acknowledge and consider women’s and men’s specific needs. One phone-based messaging DHI to deliver MNCH communication in rural Malawi found that it was feasible to provide health information to low-literate clients without a mobile phone through retrievable messages on a community phone, made accessible by community volunteers [22]. Another study found that despite moderate levels of phone ownership, women were able to leverage immediate social circles to access SMS reminders to improve timely immunization in rural Kenya [23]. One review recommended disseminating messages through peer support or self-help groups, particularly in areas where women have reduced phone ownership [42]. Given men’s disproportionate ownership of mobile phones, one study suggested that community input could inform what types of messages mobile phone owners could share with non-mobile owners, such as women partners who share devices [43].

*Gender transformative* interventions address the causes of gender-based inequalities and work to transform harmful gender norms. Male engagement was the most common gender transformative approach used or recommended. One article found that male engagement in a birth preparedness application in Indonesia increased husband birth preparedness and produced positive social changes at home with increased shared responsibility [44]. Two other studies recommended some form of male engagement in DHIs to build trust with decision-makers and address harmful gender norms that hinder women’s participation in DHIs [27, 30].

An evaluation found that mHealth for Safer Deliveries, a program that reached over 13,000 pregnant women in Zanzibar, was successful largely due to the integration of mHealth approaches into a broader community-based intervention that effectively linked institutions and communities to overcome barriers [32]. The intervention effectively empowered CHWs to reach rural women and connect them with appropriate services.

Overall, gender-responsive and transformative interventions were rare, with most DHIs falling into the gender-blind category, limiting their ability to address structural inequities in access and outcomes.

## Discussion

Among the papers included in the broader review, a small subset considered gender, despite it being a critical factor influencing the success of DHIs. When gender was mentioned, it was primarily discussed in terms of barriers, rather than as a factor integrated into the design or evaluation of the interventions. Additionally, gender-responsive DHI approaches (i.e. those that actively acknowledge, consider, and address gender-based differences and inequalities) are relatively new and may not yet be fully represented in the existing literature, making it even less likely for them to be included in the current body of reviews.

Contrary to expectations, none of the reviews explored unintended consequences of DHIs for women and girls, which we know exist through related qualitative literature not captured by our methodological approach. These include exacerbating the digital gender divide, increasing gender-based violence, and reinforcing existing gender inequities in healthcare access [16, 45, 46]. This may be linked to the limited use of gender measurements in global health data sets and program evaluations [47]. Many proxies for gender were used, including but not limited to literacy, employment, and socio-economic status. But other measures that capture gender inequalities in key socioeconomic and health indicators exist (i.e. Gender Development Index, Global Gender Gap Index, Gender Inequality Index, and the survey-based women’s empowerment index (SWPER)) and should be used to better understand the impact of DHIs on gender equity domains [48].

**Fig. 4:**
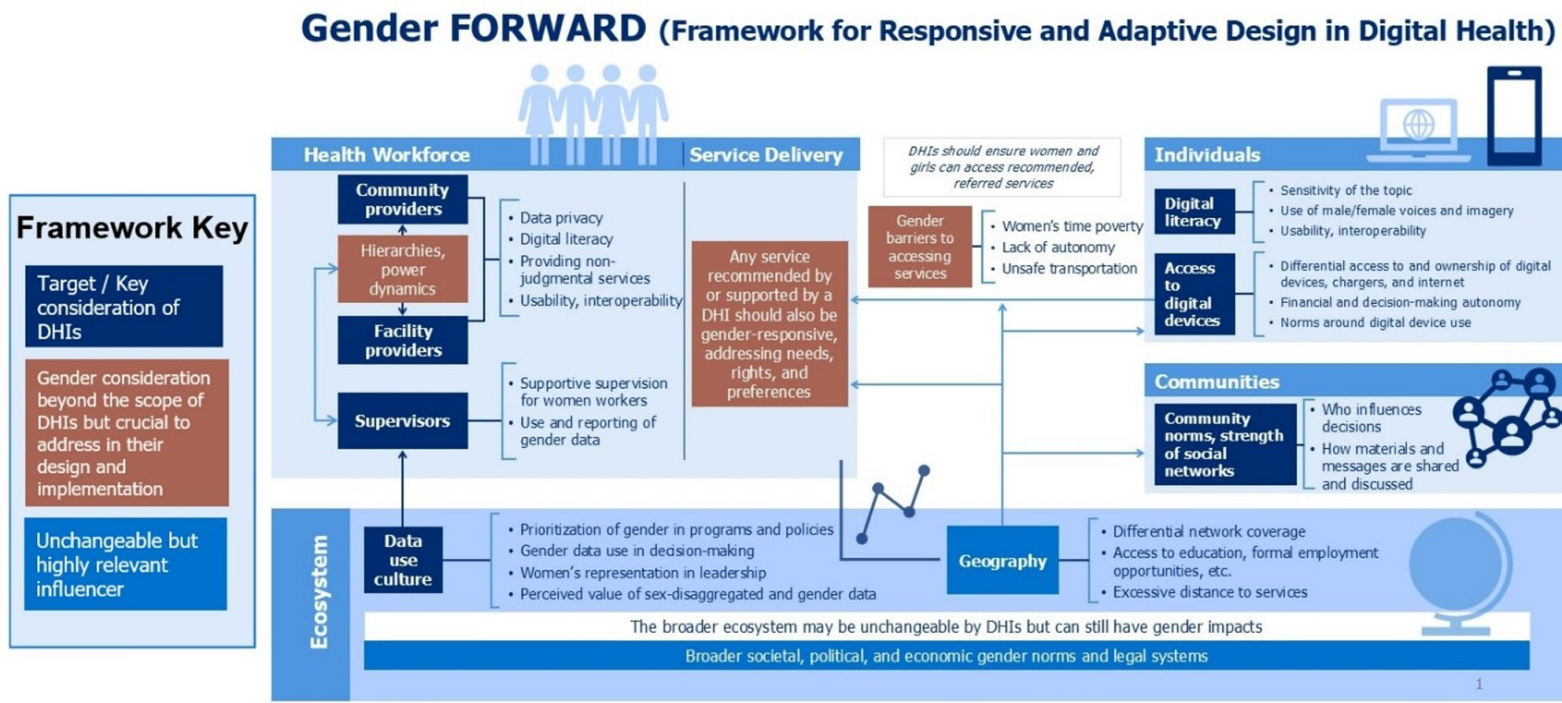
Gender FORWARD (Framework for Responsive and Adaptive Design in Digital Health)

These gaps in use of sex-disaggregated data and gender measures illuminate important opportunities for gender integration across the life cycle of DHIs. Results from this umbrella review of reviews informed the development of a framework designed to support implementors in enhancing gender-responsive design and implementation of DHIs. The Gender FORWARD (Framework for Responsive and Adaptive Design in Digital Health) framework, illustrated in Fig. 4, is organized by the target of DHIs (i.e. individuals, health workforce, and DHI ecosystem) and centers health service delivery systems. Gender FORWARD is the first framework to explicitly integrate gender across these domains to improve the gender responsiveness of DHIs.

This framework builds on previous gender-focused approaches in digital health by incorporating elements from established frameworks such as gender integration continuum. Gender FORWARD provides a tailored approach to the unique challenges and opportunities presented by DHIs and provides a targeted focus on the intersection of gender and digital health across multiple levels of the health system. This framework further expands on previous work by emphasizing the importance of adaptability and responsiveness in DHI design and implementation.

Recommendations for implementors to design and implement gender-responsive DHIs are embedded in each section below and are summarized in Table 2. Given the interconnectedness of the gender dimensions shown in the framework, there is no specific entry-point. However, implementors, researchers, and funders could consider starting at the DHI target (i.e. individuals or health workforce) and following pathways of influence from there.

### DHIs targeting individuals

Should account for women and girls’ differential literacy, digital literacy, and access to digital services. This could mean tailoring interventions to use imagery instead of text where literacy levels are lower or rely on women’s voices versus men’s voices based on preference and appropriateness. The use of gender-sensitive imagery and voices may be particularly important for sensitive topics including family planning or sexual health. Given the gender digital divide, women also tend to have lower levels of digital literacy, though this can vary by age, education, and geography. DHIs should be designed with usability considerations, ensuring that applications are fit for purpose, and preferably interoperable with other commonly used features such as mobile banking. Interoperability ensures usability, confidentiality, and safety, and reduces gender disparities by streamlining access to health services and enhancing women’s financial and healthcare decision-making power.

Results from this umbrella review of reviews also highlighted women’s differential access to and ownership of digital devices and necessary accessories such as for charging. This can be related to reduced financial autonomy and decision-making. The literature also describes gendered norms around digital device use which can govern the frequency of women’s access to devices, duration of that access, and specific times of day in which access is appropriate (i.e. in the evening following other home care responsibilities). Interventions may need to utilize accessible technologies such as basic mobile phones to enhance reach, ensure affordability and accessibility to mitigate financial barriers, and tailor interventions to consider socio-cultural barriers to improve adoption.

Communities can also influence the reach and effectiveness of DHIs. One study highlighted the importance of strong social networks within communities which were leveraged to share information from DHIs when women missed timed calls or wanted access to recorded information [22]. We also know that community leaders and religious leaders can influence the uptake of intervention messages, particularly if that messaging doesn’t align with historical norms related to sensitive topics [49–51]. Given that most leaders globally, across levels of leadership, are men, this suggests the importance of male engagement to enhance the reach and effectiveness of DHIs. In their review, Safieh et al. noted that the involvement of male partners in family planning decision-making is ‘significant’ as an outreach strategy [52]. Mildon and Sellen (2019) also found that male engagement helped to build trust with household decision-makers [42].

### Gender responsive service delivery and access

Many DHIs provide referrals or suggestions for women to seek health services from the health system. Women face distinct barriers to accessing services including but not limited to time poverty (their chronic lack of discretionary time due to unequal burdens of unpaid care, domestic work, and competing responsibilities), lack of financial autonomy, and limited, unsafe, and/or unaffordable transportation [53–55]. Geography can also determine access to digital services, access to health services, and access to other key determinants which influence literacy and financial autonomy (i.e. access to education and formal employment opportunities) [6, 56–58]. DHIs likely do not have influence on women and girls’ access to these services but should consider their unique influence based on context.

Further, any service recommended or supported by a DHI should also be gender responsive. For example, when referring women to family planning services, implementors should consider if multiple options are available (i.e. Long-acting reversible contraceptives, pills, condoms, etc.) including discontinuation. Privacy for women receiving sensitive services and respectful care are also important gender considerations as many women experience shaming from health workers [59, 60].

### DHIs targeting the health workforce

These interventions may target different levels of the health workforce including community providers (i.e. community-health workers, lady health workers, and other similar cadres), facility-based providers (i.e. nurses and doctors), and supervisors and managers. Gender power hierarchies exist between these groups where community providers are more often women, less well-paid (or not paid at all), and less educated than facility-providers. Supervisors have historically been majority men who do not always consider the unique needs of women workers or promote their advancement. These hierarchies may not be fully addressed by DHIs but should be considered by implementors, especially in interventions where community-based providers are being equipped with digital devices. In fact, DHIs designed and implemented according to the Gender FORWARD framework may be used to challenge, rather than navigate or work within existing inequitable health systems.

Digitization can impose greater responsibilities and workloads on community health workers like data collection, reporting, and patient monitoring. These tasks are often added without additional remuneration or structural support [16, 61]. Without careful consideration, digitization could exacerbate existing gender inequities by increasing the burden on women community health workers without improving their working conditions, decision-making power, or career progression. Implementors can address these challenges by ensuring fair compensation, equitable access to technology and training, and mechanisms for CHWs to participate in decision-making about digital health interventions. One recent study highlighted how CHW participation in the design process informed the integration of health worker payments into an existing community health worker application. Researchers found that this interoperability enhanced efficiency, accuracy, and gave health workers a greater sense of control [62].

DHIs targeting providers should plan for issues related to data privacy (i.e. use of personal devices and device-sharing at home), provider digital literacy, and the usability and interoperability of applications. Given many women’s reported experiences of sub-optimal quality care related to stigma and judgement, DHIs should also support the provision of non-judgmental and gender-sensitive services.

Effective supportive supervision can lead to increased health worker productivity and build trust between health workers and the health system. Women health workers face numerous challenges related to gender and supervision, which are deeply rooted in systemic inequalities and power imbalances within healthcare settings. These challenges are multifaceted, affecting their professional development, personal well-being, and work-life balance. Women health workers experience gender discrimination, including being overlooked for promotion and leadership positions [63]. Women workers continue to face a pay gap, earning less than their male counterparts, despite making up nearly most of the health workforce [64]. Supervisors who prioritize gender equity can advocate for fairness and inclusion at multiple levels: by supporting women health workers, promoting organizational changes that address gender disparities, and ensuring the use of sex-disaggregated data to inform decision-making.

### DHIs targeting the digital ecosystem

While the broader ecosystem in each country will be influenced by societal, political, and economic gender norms and legal systems which are unchangeable by DHIs, these should be well-mapped and understood prior to program design. These factors may influence broader system support of DHIs and the uptake of such DHIs among target populations.

Data collected by DHIs are often fed into larger data systems which can inform policies and programs [65, 66]. At a minimum such data should be sex-disaggregated to account for men and women’s differential uptake and use of services. More nuanced gender-related data collection and analysis is preferred when data captures women and girl’s access to resources, distribution of labor, and decision-making. Finally, women’s representation in leadership has been shown to influence the prioritization of gender integration in policies and programs which can positively influence norms in the broader ecosystem [67].

**Table 2:** Gender recommendations for DHI implementation and design

DHIs targeting individuals • Account for women and girls’ differential literacy, digital literacy, and access to digital services when designing DHIs. • Design DHIs with usability considerations, ensuring that applications are fit for purpose and preferably interoperable with other commonly used features such as mobile banking (for example). • Utilize accessible technologies such as basic mobile phones to enhance reach, ensure affordability and accessibility to mitigate financial barriers, and tailor interventions to consider socio-cultural barriers to improve adoption. • Engage men during outreach initiatives to enhance reach and effectiveness. • When referring women to services through DHIs, consider whether those services are available and responsive to women’s needs, rights, and preferences.
DHIs targeting the health workforce • Consider and account for existing hierarchies in the workforce (i.e. supervisors, healthcare practitioners, community-based providers). • Ensure fair compensation, equitable access to technology and training, and mechanisms for community-health workers to participate in the decision-making. • Plan for issues related to data privacy, provider digital literacy, and usability and interoperability of applications. • Promote effective supportive supervision.
DHIs targeting the digital ecosystem • Prior to program design, conduct a gender situational analysis to understand the societal, political, and economic gender norms and legal systems. • Ensure data is sex-disaggregated to account for men and women’s differential uptake and use of services. • Capture additional gender data whenever possible.

This framework provides a novel resource for implementors and can serve as a gender guide in program design and delivery. Implementors can use the framework to conduct a gender needs assessment which will help identify whether their programs account for the diverse needs, preferences, and constraints experienced by women, men, and gender minority individuals. The framework can also help identify opportunities for gender-transformative approaches that challenge and shift harmful norms. For example, the framework could inform the development of gender-focused monitoring checklists to track whether the intervention is reaching and benefiting intended groups as planned. Building on that tool, implementors could identify and pilot specific gender-transformative strategies such as engaging men or incorporating content that challenges harmful gender norms. It can also be used to guide regular consultations with stakeholders to identify emerging barriers and opportunities for gender-responsive programming.

Funders supporting gender integration into DHIs can use the framework to ensure new projects adequately consider gender in their design, results framework, and learning products. By requiring that grant applications include a gender integration plan based on the framework, funders can ensure that projects carefully detail how gender considerations will be incorporated at every stage. This includes embedding gender responsive indicators, requiring sex-disaggregated data collection (at a minimum), and support implementation research that captures gendered impacts over time. Funders can also create capacity-building opportunities for grantees and implementors on gender-responsive design and evaluation, using the framework as a training tool.

Researchers conducting monitoring and evaluation can use Gender FORWARD in conjunction with gender-responsive M&E approaches to enhance the usability of findings and their uptake by decision-makers [68]. Morgan et al.’s gender-responsive M&E frameworks argues that sex or gender specific and sex or gender disaggregated data are entry points; the integration of needs, rights, and preferences and gender power relations and systems enhances gender responsiveness [68]. This includes embedding qualitative approaches which allow for the exploration of topics in more depth and provide the complexity needed when addressing systems of power. By using key-informant interviews, focus group discussions, and observations, implementors may be better able to identify potential unintended consequences and make adaptations to avoid or mitigate them. Gender-responsive M&E can therefore enhance the utility of Gender FORWARD.

### Limitations

This review is subject to certain limitations. Reviews were only included if they reported quantitative outcomes. While this approach allows us to highlight measures of effectiveness, it has likely led to missing literature on gender barriers and unintended outcomes. The exclusion of qualitative studies may have also led to the omission of important findings related to gender norms, power, distribution of labor, and gender-transformative interventions. Qualitative research often provides rich, contextualized insights into how DHIs influence or interacts with entrenched gender roles, cultural practices, and social hierarchies. This review may therefore have missed nuanced perspectives on how gender norms are reproduced or challenged, the mechanisms of power dynamics within households and communities, and the lived experiences of intervention participants, especially those belonging to marginalized groups. Additionally, qualitative evidence is vital for understanding the complexity and effectiveness of gender-transformative interventions, including process-related factors and unintended consequences that may not be captured by quantitative metrics.

While we discuss the importance of using an intersectional lens, such intersections with gender and age, race, disability, socio-economic status, geography, etc. were rarely explored in our data. The inclusion of qualitative data may also have enhanced our ability to integrate intersectional considerations.

Some outcomes were also challenging to define, and a lack of standardized outcomes may have resulted in a restricted ability to identify reviews for gender analysis.

Many of the included studies are from South Asia, particularly India. Further, reviews were only included if they were published in English. This may limit generalizability to other LMIC regions, such as West or Central Africa or Latin America.

The limited quantity and quality of available evidence on gender responsive and gender transformative interventions may also influence the applicability of the framework. However, the use of existing gender transformative frameworks to guide analysis is an important strength. Further, by using the framework to inform stakeholder consultations and iterative feedback processes, the framework can become increasingly responsive to changing gender norms.

## Conclusion

Our review highlights the significant barriers women face, including limited phone ownership, financial dependence, and socio-cultural norms, which impede their access to DHIs. Gender Framework for Responsive and Adaptive Design in Digital Health (FORWARD) is the first tool of its kind that can be used to support gender integration activities to enhance the gender-responsiveness, effectiveness and equity of DHIs. Gender FORWARD should be actively integrated into the design, monitoring, evaluation, and scale of DHIS across stings. This framework provides practical guidance for implementors to design and deliver DHIs that are not only effective but also equitable, ensuring that gender considerations are appropriately integrated into every stage of the intervention. The tool can similarly be used by researchers to guide gender responsive monitoring and evaluation, by funders to ensure gender-responsive interventions, and by policy makers institutionalizing gender responsive strategies. Future research should include validation of the framework with implementors and testing the framework in practice, ensuring qualitative measures are used to capture gender-related barriers and outcomes. Continuous feedback from practitioners, end-users, and affected communities can inform updates to the framework, making it increasingly responsive to evolving gender norms and intersectional inequities, and support its use in mitigating unintended consequences. By prioritizing iterative learning, gender FORWARD can drive transformative change in gender-responsive digital health programming worldwide.

## Data Availability

All data generated or analyzed during this study are included in this published article.
